# Hair cortisol-a method to detect chronic cortisol levels in patients with Prader-Willi syndrome

**DOI:** 10.1186/s12902-020-00646-w

**Published:** 2020-11-10

**Authors:** Hasanain Hamid Shukur, Yolanda B. de Rijke, Elisabeth F. C. van Rossum, Laith Hussain-Alkhateeb, Charlotte Höybye

**Affiliations:** 1 Department of Molecular Medicine and Surgery, Karolinska Institute, Stockholm, Sweden; 2 Department of Clinical Chemistry, Erasmus MC, University Medical Center Rotterdam, Rotterdam, The Netherlands; 3 Department of Medicine, Division of Endocrinology, Erasmus MC, University medical Center Rotterdam, Rotterdam, The Netherlands; 4 Obesity Center CGG, Erasmus MC, University Medical Center Rotterdam, Rotterdam, The Netherlands; 5 School of Public Health and Community Medicine, Institute of Medicine, Sahlgrenska Academy, University of Gothenburg, Gothenburg, Sweden; 6 Patient Area Endocrinology and Nephrology, Inflammation and Infection Theme, Karolinska University Hospital, Stockholm, Sweden

**Keywords:** Prader-Willi syndrome, Hair cortisol, Chronic stress, Obesity

## Abstract

**Background:**

Prader-Willi syndrome (PWS) is a multisymptomatic, rare, genetic, neurodevelopmental disorder in adults mainly characterized by hyperphagia, cognitive dysfunction, behavioral problems and risk of morbid obesity. Although endocrine insufficiencies are common, hypocortisolism is rare and knowledge on long-term cortisol concentrations is lacking. The aim of this study was to evaluate long-term cortisol levels in PWS by measurements of hair cortisol.

**Methods:**

Twenty-nine adults with PWS, 15 men and 14 women, median age 29 years, median BMI 27 kg/m^2^, were included. Scalp hair samples were analyzed for cortisol content using liquid-chromatography tandem-mass spectrometry. In addition, a questionnaire on auxology, medication and stress were included. For comparison, 105 age- and sex-matched participants from the population-based Lifelines Cohort study were included as controls. The mean hair cortisol between the groups were compared and associations between BMI and stress were assessed by a generalized linear regression model.

**Results:**

In the PWS group large variations in hair cortisol was seen. Mean hair cortisol was 12.8 ± 25.4 pg/mg compared to 3.8 ± 7.3 pg/mg in controls (*p* = 0.001). The linear regression model similarly showed higher cortisol levels in patients with PWS, which remained consistent after adjusting for BMI and stress (*p* = 0.023). Furthermore, hair cortisol increased with BMI (*p* = 0.012) and reported stress (*p* = 0.014).

**Conclusion:**

Long-term cortisol concentrations were higher in patients with PWS compared to controls and increased with BMI and stress, suggesting an adequate cortisol response to chronic stress. Hair cortisol demonstrate promising applications in the context of PWS treatment and disease management.

## Background

Prader-Willi syndrome (PWS, OMIM 176270) is a rare, complex neurodevelopmental disorder with a reported incidence of approximately 1 in 15,000–30,000 newborns [1]. PWS is caused by a lack of expression of paternally inherited genes in the q11-q13 region of chromosome 15, most commonly caused by a paternal deletion or a maternal disomy [1, 2]. PWS in adults is characterized by muscular hypotonia, hyperphagia requiring restricted diet and regular physical activity to prevent the development of severe obesity [3]. Other features include a mild to moderate intellectual disability, a distinct behavioral phenotype with stubbornness, temper tantrums, obsessive and compulsive behaviors and mood instability [4, 5].

Many of the symptoms in PWS are believed to be caused by a dysfunction of the hypothalamus. Endocrine abnormalities are common including deficiencies in growth hormone, sex hormones and thyroid hormones. Concerning, the function of the hypothalamus-pituitary-adrenal (HPA) axis both normal function and partial central adrenal insufficiency have been reported [6–11].

In previous studies, different tests were used to evaluate the function of the HPA-axis. They were all based on acute variations in adrenocorticotropic hormone (ACTH) and/or cortisol, whereas information about cortisol concentrations over a longer period is lacking. Due to the intellectual disability and behavior problems in PWS it is also of great value to find other approaches for evaluating the HPA axis in PWS than the conventional tests. By measuring cortisol in hair, the average cortisol concentrations over longer periods can be assessable using an easy, non-invasive and convenient procedure [12].

The aim of this study was to examine hair cortisol in a group of adults with PWS in comparison to population-based controls. Self-reported clinical parameters from a semi-structured questionnaire were included for further evaluation.

## Methods

### Participants and procedures

Twenty-nine adults (older than 18 years of age) with PWS were enrolled in 2015 and 2016 either during routine visits to the Department of Endocrinology, Karolinska University Hospital, Stockholm, or after responding to advertisements in the journal of the Swedish PWS Association.

Data for controls were retrieved from the Lifelines Cohort Study. Lifelines is a multi-disciplinary prospective population-based cohort study examining the health and health-related behaviors of 167,729 persons living in the North of The Netherlands [13–15]. Lifelines comprises a broad range of data, including hair cortisol which was available for 266 individuals, who were used as controls in the present study [15].

### Hair processing and analysis

In summary, a hair sample (100–150 hairs) was cut from the posterior vertex of the scalp, as close to the scalp as possible. The hair sample was stored on a paper in an envelope until analysis [13]. For the analysis of hair cortisol, the proximal 3 cm of the hair (10 mg) was cut into 1 cm segments, which were washed in isopropanol, and then left to dry [13]. Methanol was used to extract cortisol and after purification, cortisol was quantified by liquid chromatography − tandem mass spectrometry (LC–MS/MS) (Waters, Milford, MA) [16]. Two standard curves were identified from ten calibration standards on each day of analysis and the functional sensitivity (lower limit of quantification, LLoQ (pg/mg)) was calculated by serial dilution from 40 mg/ml to 0.3125 mg/ml [16]. The geometric mean of hair cortisol analyses in The Lifelines Cohort study (*N* = 266) was 2.7 pg/mg [13].

### Questionnaire

The participants with PWS and/or their caretakers, filled out a questionnaire about their age, sex and anthropometric measurements. This questionnaire was not developed for this study, but has been used for multiple studies in the past.

[15]. The stress was assessed by the question: *did any stressful events occur during the last 3 months? In case there did, what happened?*

In addition, the participants were asked in a standardized manner whether they used any product containing corticosteroids in the past 3 months and the route of administration (i.e., oral, intravenous, nasal, topical, inhaled, joint injections or others).

Data retrieved from the Lifelines Study for the controls included information on the subjects’ age, sex, anthropometric and stressful life events. Life events were evaluated using the Dutch version of the List of Threatening Experiences (LTE), including occurrence of twelve major life events in the past 12 months [13].

### Matching process and statistical analysis

The PWS patients were categorized into four age-groups [(18–25 years), (26–35 years), (36–45 years) and (46–60 years)], resulting in a fairly balanced age-sex groups for each of the approximately 10 years intervals. Subjects from each group of PWS patients were matched for age and sex to a total of 105 subjects from the corresponding control groups, in a 1:5 ratio. However, due to low number of controls for males in the 18–25 age-group (9 adults with PWS, 23 matched-controls) matching was made in a ratio of 1:2.5 for this particular sex-age group. The matching was programmed using the “seed” command in STATA 15.0 to ensure a random selection of subjects.

Results are presented as mean ± SD. For comparison between the groups, Student’s t-test was used. Mean hair cortisol was considered to best capture the clinical reality with large variations in hair cortisol and a generalized linear regression model was used to examine the association of crude levels and levels adjusted for BMI and stress as potential confounders. The Akaike Information Criteria (AIC) test was used to confirm best fit model where by both BMI and reported stress were retained in the model, as they predicted the model well. Statistical significance was set at *p* < 0.05.

## Results

Twenty-nine adults (15 men and 14 women) with confirmed PWS participated. Mean (SD) for age was 33.4 years (12.7) in the patients with PWS and 42.1 (11.6) years in the controls. By virtue of the matching process based on the sex-age groups, there were no significant differences between the patients with PWS and controls with respect to age and sex.

In the lifelines Cohort Study median BMI was 26 kg/m2, 42% were overweight and 19% obese [15]. Twelve percent reported use of steroids the past 3 months and 58% reported at least one life event during the past 12 months [15].

Individual hair cortisol levels in patients with PWS are displayed in Table 1. Six patients with PWS had hair cortisol levels > 10 pg/mg and all but two of them reported occurrence of stressful situations. Seven reported use of steroids without hair cortisol being suppressed (hair cortisol 2.0 to 39.1 pg/mg) and all but one reported stress.

**Table 1:** Characterization of 29 patients with Prader-Willi syndrome, as a heat map table, sorted according to the levels of hair cortisol

Patient code	Hair cortisol pg/mg	BMI	Stress	Corticosteroids
**26**	105,6	40,0	**yes**	**0**
**12**	90	26,0	**yes**	**0**
**18**	39,4	28,0	**yes**	**Inhalation**
**1**	36,1	32,0	**no**	**Cream ointment for eczema**
**7**	12,5	26,0	**yes**	**0**
**14**	11,4	22,0	**no**	**0**
**10**	10	35,0	**no**	**0**
**6**	8,5	20,0	**yes**	**Ointment**
**5**	7,7	20,0	**yes**	**Ointment**
**28**	7,6	45,0	**no**	**0**
**21**	5,6	27,0	**no**	**0**
**20**	3,3	27,0	**no**	**0**
**13**	3,2	28,0	**yes**	**Very few ointment cream**
**27**	3	39,0	**Yes**	**Nasal spray**
**8**	2,7	23,0	**no**	**Very few ointment cream**
**11**	2,1	24,0	**no**	**0**
**19**	2,1	23,0	**yes**	**0**
**3**	2	48,0	**no**	**0**
**4**	1,9	32,0	**yes**	**0**
**25**	1,9	32,0	**yes**	**0**
**23**	1,8	23,0	**yes**	**0**
**29**	1,5	24,0	**yes**	**0**
**9**	1,4	24,0	**yes**	**0**
**24**	1,3	31,0	**no**	**0**
**22**	1,3	39,0	**no**	**0**
**2**	1,3	33,0	**no**	**0**
**15**	1,3	37,0	**no**	**0**
**16**	1,3	25,0	**no**	**0**
**17**	1,3	18,0	**no**	**0**

**Fig. 1:**
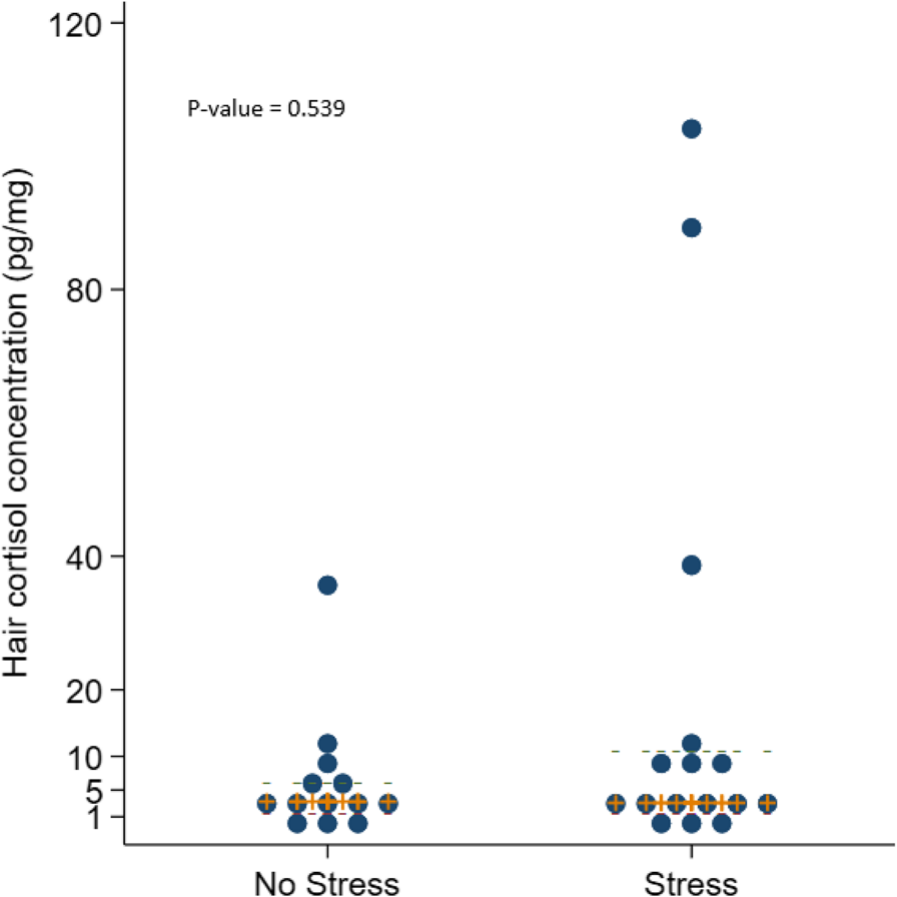
Swarm plot of hair cortisol in 29 patients with Prader-Willi syndrome with or without stress, displaying the median (IQR) and included a *p*-value

Thirteen other adults with hair cortisol of 10 pg/mg and below had not experienced any stress, and the remaining ten adults reported different kinds of stress that did not result in high levels of hair cortisol. Six patients with hair cortisol < 1.3 pg/mg did not report any stress or use of glucocorticoids. Figure 1 shows the hair cortisol in patients with PWS with and without reported stress.

There was a large variation in hair cortisol levels in the patients with PWS. Mean hair cortisol in all patients with PWS was 12.8 ± 25.4 pg/mg compared to 3.8 ± 7.3 pg/mg in the controls (*p* = 0.001). When the two high readings were excluded mean hair cortisol in the PWS group was 6.43 ± 9.66 pg/mg. In the crude regression model, hair cortisol in patients with PWS was significantly higher compared to the controls (β = 1.21; 95% CI [0.44–1.98]) (*p* = 0.002) and remained higher after adjusting for BMI and reported stress (β = 0.84; 95% CI [0.11–1.56], *p* = 0.023) (Table 2).

**Table 2:** Crude and adjusted regression Coefficients and 95% Confidence intervals (CI) using a linear regression model for hair cortisol (pg/mg), in 29 patients with Prader-Willi syndrome (PWS) and 105 age- and sex-matched controls

	Crude		Adjusted^b^	
	Regression Coefficients β (95% CI)	*P*-value	Regression Coefficients Β (95% CI)	*P*-value
**Controls** **PWS**	Reference 1.21 (0.44–1.98)	0.002	Reference 0.84 (0.11–1.56)	0.023
**PWS** **BMI** kg/m^2 a^	0.05 (0.02–0.09)	0.002	0.04 (0.01–0.08)	0.012
**PWS** **Stress** **Yes**	1.16 (0.41–1.91)	0.002	0.90 (0.19–1.60)	0.014

^a^BMI (Body Mass Index)

^b^adjusted for BMI and stress

Using the same linear regression model in patients with PWS, hair cortisol increased with BMI (β = 0.05; 95% CI [0.02–0.09], *p* = 0.002, which remained after adjustment for stress (β = 0.04; 95% CI [0.01–0.08], *p* = 0.012) (Table 2). Likewise, hair cortisol increased with stress (β = 1.16; 95% CI [0.41–1.91], p = 0.002), which remained after adjustment for BMI (β = 0.90 (95% CI [0.19–1.60], *p* = 0.014) (Table 2).

## Discussion

In this study of 29 adults with PWS, mean hair cortisol levels were significantly higher than hair cortisol in 105 sex and age matched population-based controls. Hair cortisol increased with BMI and reported stress in patients with PWS.

The stress hormone cortisol, produced in the adrenals, is important in maintaining body homeostasis by modulating neuroendocrine functions, the sympathetic nervous system and the immune function. Cortisol levels are regulated by activity in the hypothalamus and pituitary and secreted in a pulsatile circadian rhythm with increased secretion in response to stress. Cortisol is commonly measured in blood, and saliva, but these methods only reflect the time point at which the sampling is done or when measured in urine the past 24 h’ cortisol production. Multiple samplings are often needed. However, during the past decade, the measurement of hair cortisol has emerged as a non-invasive method to estimate the cortisol concentrations over months [6, 14, 17]. Hair grows at a relatively stable rate of approximately 1 cm/month, and cortisol accumulates in hair. Thus, cortisol in hair retrospectively represents the average cortisol level over the corresponding period. There is no discomfort associated with collection of the hair sample and no special storage precautions are required for the samples. This is an attractive procedure, especially in a population such as PWS where the psychological and behavioral features might complicate advanced sampling procedures. We found that collection of the hair samples worked out well in our PWS cohort, although some men could not participate because of too short hair for sufficient analyses. In addition, four patients declined participation because they felt that even the small hairless spot on the scalp after cutting the hair would stigmatize them further, although the sampling spot at the back of the scalp is usually too small to be visible afterwards. In our cohort of patients with PWS, hair cortisol levels were clearly increased in patients who had reported significant stressful events. This is in accordance with several studies showing that the content of cortisol in hair is higher in individuals exposed to stress and stress-related disorders [12, 17–24]. Some of the patients in the present study with normal to low normal hair cortisol reported nonspecific stressful situations, but the analysis of hair cortisol was probably not sensitive enough to detect minor or short-lived alterations of the HPA-axis [25].

Cortisol levels have been found to correlate positively with waist to hip ratio, waist circumference and BMI [19, 26–30] and to be higher in obese individuals compared to normal-weight people [28, 29]. To retain all patients with PWS in the statistical analyses, matching for BMI between patients with PWS and controls was not done since some severely obese patients with PWS could not be matched with controls. However, similar to previous studies, we found an increase in hair cortisol with BMI in the patients with PWS [30].

It is well known that some patients with PWS suffer from diseases secondarily to obesity, such as diabetes, cardiovascular diseases, respiratory diseases and arthrosis [31], and PWS is associated with a decreased life expectancy, with an annual death rate reported to be as high as 3% [32, 33]. Nevertheless, a number of reported deaths were unexpected and unexplained, and it has been speculated if central adrenal insufficiency might have been the cause of some of the deaths [6]. While the ACTH stimulation test provides information about the maximum response of the adrenal gland under stress hair cortisol concentrations provide information about average cortisol concentrations over a longer period. Our results warrant for larger and potentially randomized studies, but measurement of hair cortisol in PWS is attractive due to its non-invasive and simple performance. It is also an advantage that hair cortisol gives information on long-term cortisol exposure and might in the future be a baseline test for evaluation of the adrenal function and consideration for further evaluation.

### Limitations

PWS is a rare disease, and the number of participants in the present study was too small for more complex analyses and comparisons with the control group. Furthermore, detailed information on use of steroids was not realistic to obtain and for robust information a prospective study would be necessary. Hair cortisone was not analyzed, but as hair cortisol and hair cortisone strongly correlate [30], the added value of measuring cortisone for the purpose of the present study would probably be limited.

Another limitation in our study is the assessment of stress, which in both groups was interpreted from reported stress during different duration of time and different questionnaires, which potentially might have affected the results. Moreover, in the PWS group most of the questionnaires were answered by the patients’ caretakers and not directly by themselves. The questions on stress were not validated in patients with PWS but they were adapted to the capacity of this group of patients and therefore very simple to answer and the answers were confirmed by the caregivers. However, this is a pilot study and for the purpose of our study we were interested in whether stressful situations at all would elicit an increase in cortisol. Finally, patients with PWS who were able and willing to participate, might represent a selected group of quite healthy and well-functioning adults with PWS.

## Conclusion

In conclusion, in our adult patients with PWS the variation in hair cortisol was large. The mean cortisol level was higher than in the age and sex matched population-based controls. Increased BMI and reported stress were both associated with increased hair cortisol levels. The function of the HPA axis in PWS is an ongoing issue and there is a need for further investigations. Among these to study the long-term cortisol exposure and the effect of stress. The present study is the first to analyze hair cortisol in PWS, and our results suggest an adequate long-term cortisol level and cortisol response to chronic stress in PWS. Hair cortisol might become a useful tool to guide the monitoring and management efforts for adults with PWS, including follow-up of comorbidities.

## Data Availability

The data that support the findings of this study are available on request from the corresponding author. The data are not publicly available due to privacy or ethical restrictions. Data from controls are kept by the Lifelines Cohort study, the Netherlands. The permission for access to the raw data from the Lifelines cohort study was granted by the Lifelines’ research office.

## Abbreviations

PWS: Prader-Willi syndrome
HPA axis: Hypothalamus pituitary adrenal axis
BMI: Body mass index
ACTH: Adrenocorticotropic hormone
